# Artificial intelligence in respiratory care

**DOI:** 10.3389/fdgth.2024.1502434

**Published:** 2024-12-23

**Authors:** Manjush Karthika, Jithin K. Sreedharan, Madhuragauri Shevade, Chris Sara Mathew, Santosh Ray

**Affiliations:** ^1^ Faculty of Medical and Health Sciences, Liwa College, Abu Dhabi, United Arab Emirates; ^2^ Department of Respiratory Therapy, University of Doha for Science and Technology, Doha, Qatar; ^3^ Department of Training Programs, Chest Research & Training Pvt Ltd., Pune, India; ^4^ Faculty of Engineering and Computing, Liwa College, Abu Dhabi, United Arab Emirates

**Keywords:** artificial intelligence, machine learning, deep learning, respiratory care, mechanical ventilation

## Abstract

The evolution of artificial intelligence (AI) has revolutionised numerous aspects of our daily lives, with profound implications across various sectors, including healthcare. Although the concept of AI in healthcare was introduced in the early 1970s, the integration of this technology in healthcare is still in the evolution phase. Despite barriers, the current decade is witnessing an increased utility of AI into diverse specialities of the medical field to enhance precision medicine, predict diagnosis, therapeutic results, and prognosis; this includes respiratory medicine, critical care, and in their allied specialties. AI algorithms are widely studied in areas like mechanical ventilation, sleep medicine, lung ultrasound, and pulmonary function diagnostics and the results are found to be promising. The quality of patient care and safety can be greatly enhanced if respiratory care professionals fully understand the concept and importance of AI, as they are already incorporating various aspects of this technology into their clinical practice. Awareness of AI in the clinical field is essential during this phase; hence, it is desirable to establish widely accepted standards presented in a clear and accessible language. This article aims to describe the existing and prospective role of AI in the field of respiratory care and allied areas.

## Introduction

Artificial intelligence (AI) refers to the simulation of human intelligence, including critical thinking, perception, reasoning, learning, planning, and predicting, using systems or machines (1). John McCarthy, an American computer and cognitive scientist, coined the term “artificial intelligence” in 1956, describing it as “the science and engineering of making intelligent machines (2).” AI enables technology to perform tasks intelligently and act rationally like humans without needing explicit programming.

AI is classified into three categories: First, artificial narrow intelligence (narrow or weak AI) is goal-oriented and designed for specific tasks. While these systems are considered intelligent, they do not mimic human intelligence. These systems simulate human behaviour based on predefined parameters, for example, virtual assistants on smartphones and email spam filters. Second, artificial general intelligence (strong or deep AI) are machines that mimic human intelligence, potentially solving problems similarly to humans. Researchers are still working to achieve this level of machine intelligence, characterised by hypothesis testing, imagination, recognition, and recall. Last, artificial super intelligence is a hypothetical concept where machines surpass human capabilities and become self-aware, as depicted in various science fiction narratives (3).

**Figure 1:**
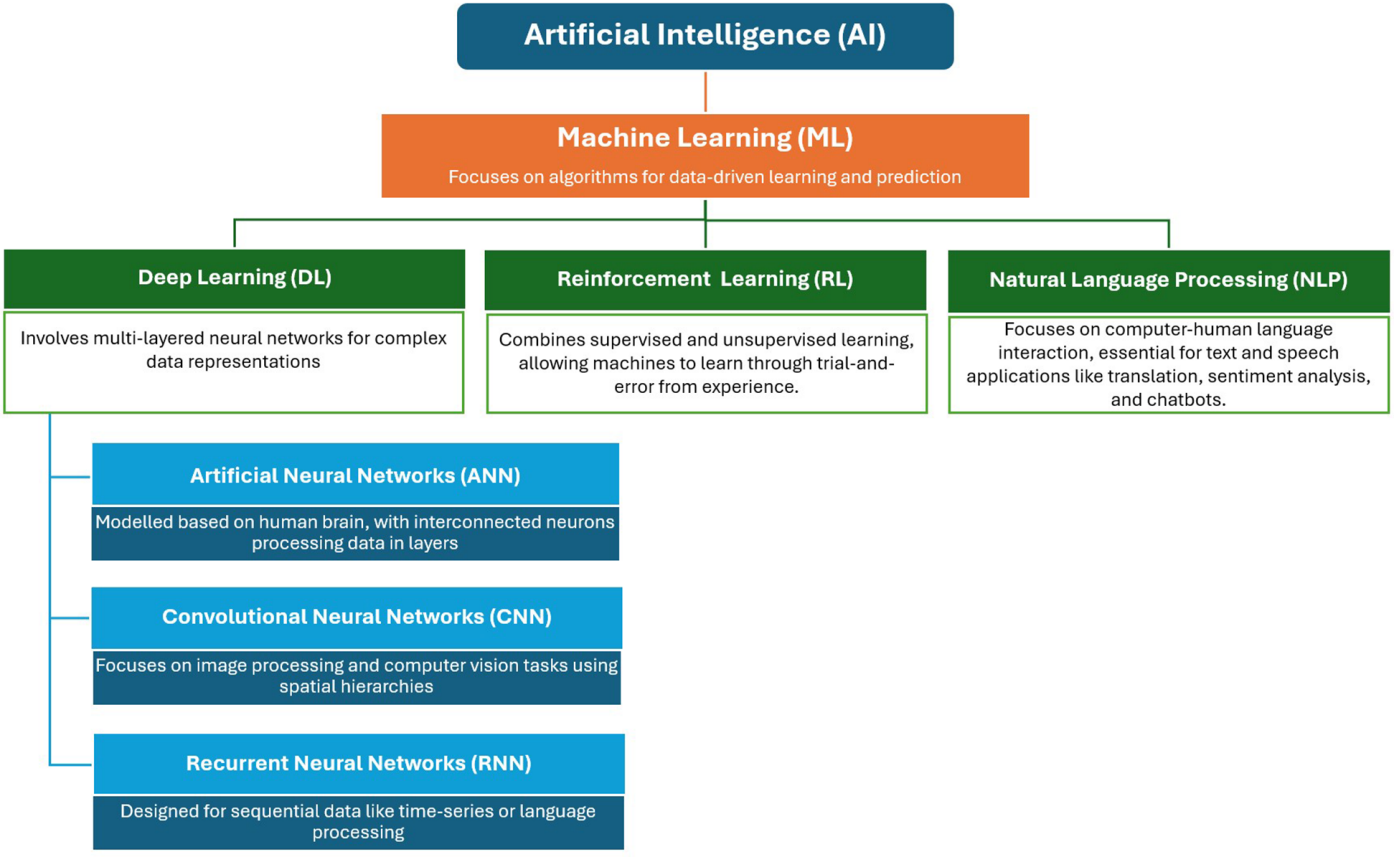
An overview of artificial intelligence.

Learning is the most crucial property of AI, reflecting the machine's ability to acquire or memorise knowledge without explicit programming. Machines learn using various approaches, such as machine learning (ML) as the broad subset of AI, with deep learning (DL) and reinforcement learning (RL) as the subsets of ML, and natural language processing (NLP) as an application area with the ML (Figure 1) (4). ML enhances performance over time by obtaining more data, empowering computer systems to “learn” independently by processing data through algorithms, detecting patterns, and making accurate predictions. Some methods include forward reasoning, backward derivation, regression, clustering, and categorisation (5, 6). DL, the subset of ML, involves artificial neural networks (ANNs) to solve problems. ANNs, composed of interconnected “neurons,” mimic the human brain's decision-making processes. DL algorithms process data through ANNs, with each layer progressively extracting information. Some commonly used DL networks include convolutional neural networks (CNNs) for image recognition, recurrent neural networks (RNNs) for sequential data, autoencoders for unsupervised learning, and generative adversarial networks for generative tasks (7, 8). RL, a subset of ML shares the characteristics of both supervised and unsupervised processes and enables machines to learn through trial-and-error using their own experiences. In RL, agents receive rewards or penalties based on outcomes, facilitating learning (4, 9). NLP refers to the specialized application of ML, focused on enabling machines to understand and process human languages. Many NLP applications, heavily rely on ML algorithms, making it a significant application area within ML.

**Figure 2:**
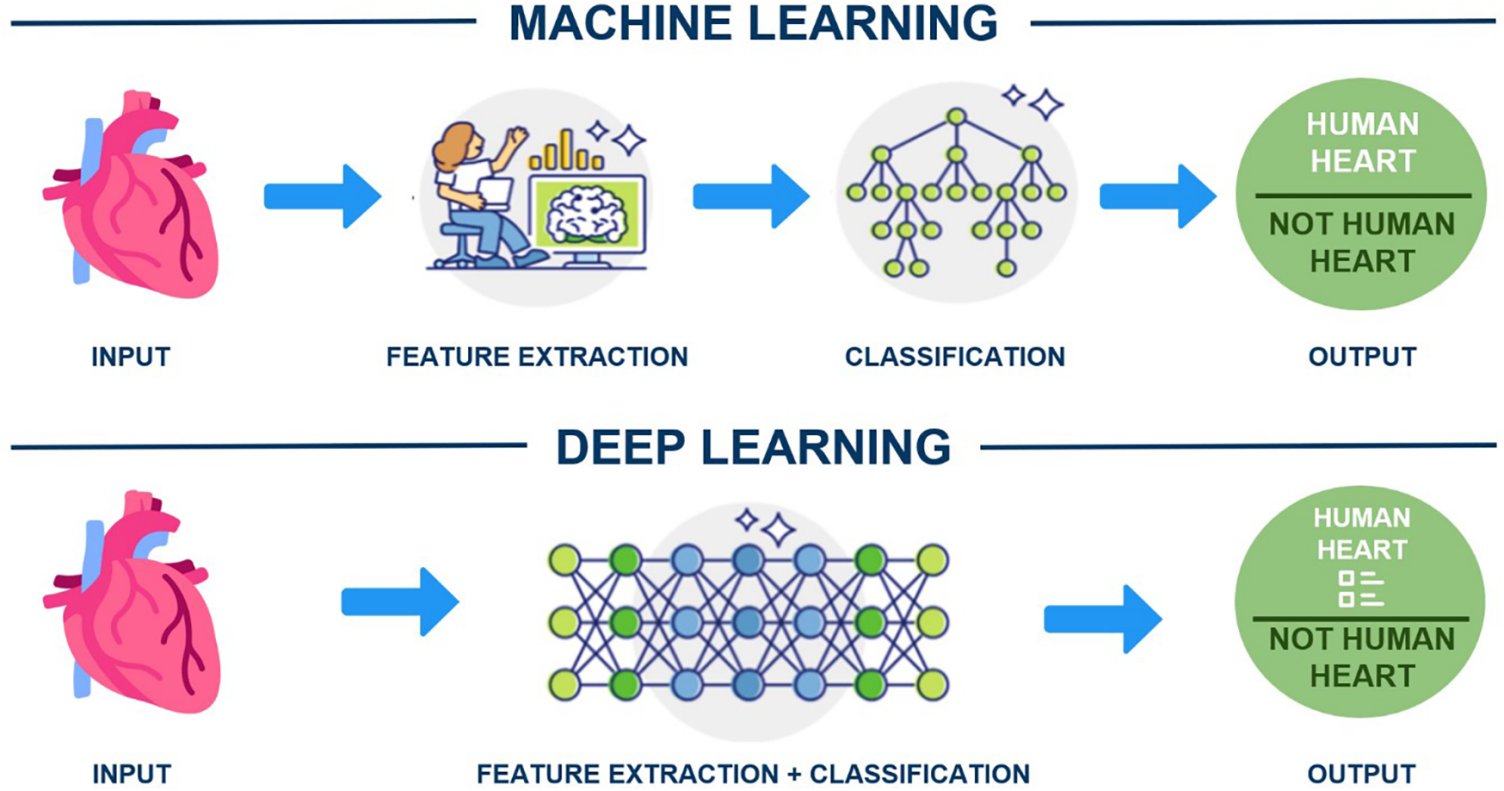
Machine learning versus deep learning.

In this technological era, clinicians should understand ML and DL tools that use structured, labelled data to make predictions. For instance, ML requires human-extracted data to identify an organ as a human heart, while DL algorithms can determine the most critical features for distinguishing organs (e.g., shape) (Figure 2). The application of AI has become increasingly widespread across industries such as healthcare, automotive, finance, transportation, and e-commerce (10). Although AI in medicine dates to the early 1970s (11), its full adoption in healthcare requires convincing evidence for both healthcare and non-healthcare communities (12). Despite significant investment, AI's implementation in healthcare remains in its early stages (13, 14). Research suggests that AI, particularly DL, could be a solution for challenges like burnout, healthcare professional shortages, cost reduction, and patient safety improvements (15). Notably, there has been an exponential rise in AI-related scholarly publications, especially after 2014 (16). AI can analyse patients' electronic health records, using DL to forecast medical events, unplanned admissions, complications, and mortality (17). Additionally, ML-powered AI can examine extensive data from genomic and clinical trials, aiding drug discovery and predicting drug efficacy and toxicity (18). The United States Food and Drug Administration and Conformité Européenne have approved over 300 AI-based software and medical devices, with most focusing on pulmonary imaging (19).

This narrative review describes the current and potential role of AI in respiratory care and related areas. The literature search for this article followed a systematic approach to identify relevant studies, articles, and reviews. Various electronic databases, such as PubMed, Scopus, Web of Science, and Google Scholar, were thoroughly searched. Key terms and phrases like “Artificial Intelligence,” “Respiratory Care,” “Machine Learning,” “Deep Learning,” “Artificial Intelligence in Respiratory Care,” “Artificial Intelligence in Critical Care,” “Artificial Intelligence in Respiratory Medicine,” and “Artificial Intelligence in Neonatology and Paediatrics” were used to conduct a comprehensive and focused search.

## AI in respiratory care

Existing literature shows that DL and ML have significant potential in managing respiratory diseases, including image analysis, decision-making, and prognosis prediction (20, 21). Notably, AI correctly interprets chest x-rays and diagnoses pulmonary tuberculosis with 95% sensitivity and 100% specificity (22). This result signifies that radiographs from remote centres can be interpreted by a centralised AI monitoring system.

Another crucial area is inhaler devices, which are vital in managing asthma and chronic obstructive pulmonary disease (COPD). However, many patients misuse them, reducing effectiveness, lowering quality of life, and increasing financial burden (23, 24). The evolution of smart inhalers with built-in electronic monitoring has been found to improve patient adherence and monitor inhalation techniques. These devices connect to a mobile app via Bluetooth, with sensors integrated into or attached to the inhaler. Smart inhalers offer features such as tracking tools, personalised alerts, and feedback based on consumption, inhalation technique, peak flow values, and questionnaire responses. These features provide patients with immediate feedback, helping them adhere to their treatment plan and manage their condition better. Literature shows that patients with COPD and asthma who use smart inhalers experience improved adherence, fewer exacerbations, and better lung function (25–27). However, obstacles to implementation include high costs and the need for appropriate patient introduction and feedback techniques (24). The scope of AI is still evolving, as these patients are monitored for years, and exacerbations can be recognised at several levels, including cellular, organ, and organismal levels. In a study, Kukreja (28) designed and assessed algorithms for diagnosing bronchial asthma using a comprehensive questionnaire, clinical data, and medical records. With reliable data training, the model with automated associative memory to the neural network algorithm achieved an accuracy of over 90% with 1% inconclusive results, and the generated mobile applications achieved 94.2% accuracy. Fernandez-Granero MA et al. (29) developed a decision tree forest model to predict symptom-based COPD exacerbations. In this study, patients' breath sounds were monitored daily for 6 months using sensor devices via telemonitoring. The data was analysed with an ML algorithm, achieving a detection accuracy of 78% and forecasting acute exacerbation of COPD approximately 4.4 days before it occurred.

AI technology is continually advancing in interventional pulmonology, helping to recognise endobronchial structures and distinguish between lung cancer subtypes by analysing appearance and texture in bronchoscopic images. Cytological rapid on-site evaluation (ROSE) is used to enhance bronchoscopic examinations by confirming specimen adequacy and accuracy in real-time. Ai D et al. (30) constructed a DL-CNN model integrated with ROSE to classify cytologic whole-slide images as malignant or benign. The resultant area under the curve (AUC) was 0.9846 for ROSE in the validation group, with a comparable accuracy of 84.6%.

Auscultation, the act of listening to sounds from within body organs using a stethoscope, is crucial in diagnosing respiratory disorders. However, auscultation is subjective, depending on device quality and clinician expertise. Digital or electronic stethoscopes are revolutionising pulmonology by enhancing accuracy and reliability in lung auscultation. These stethoscopes transform sound waves into electronic signals, which can be amplified for better listening. These signals can also be processed and converted into digital format for transmission to a computer or laptop (31). AI has shown the ability to accurately identify crackles and wheezes in breath sounds captured by various digital stethoscope devices, although some variations may occur depending on the specific device used (32). A recent study based on DL has demonstrated significant potential in analysing lung sounds, delivering more precise and reliable results compared to shallow machine learning techniques (33).

**Figure 3:**
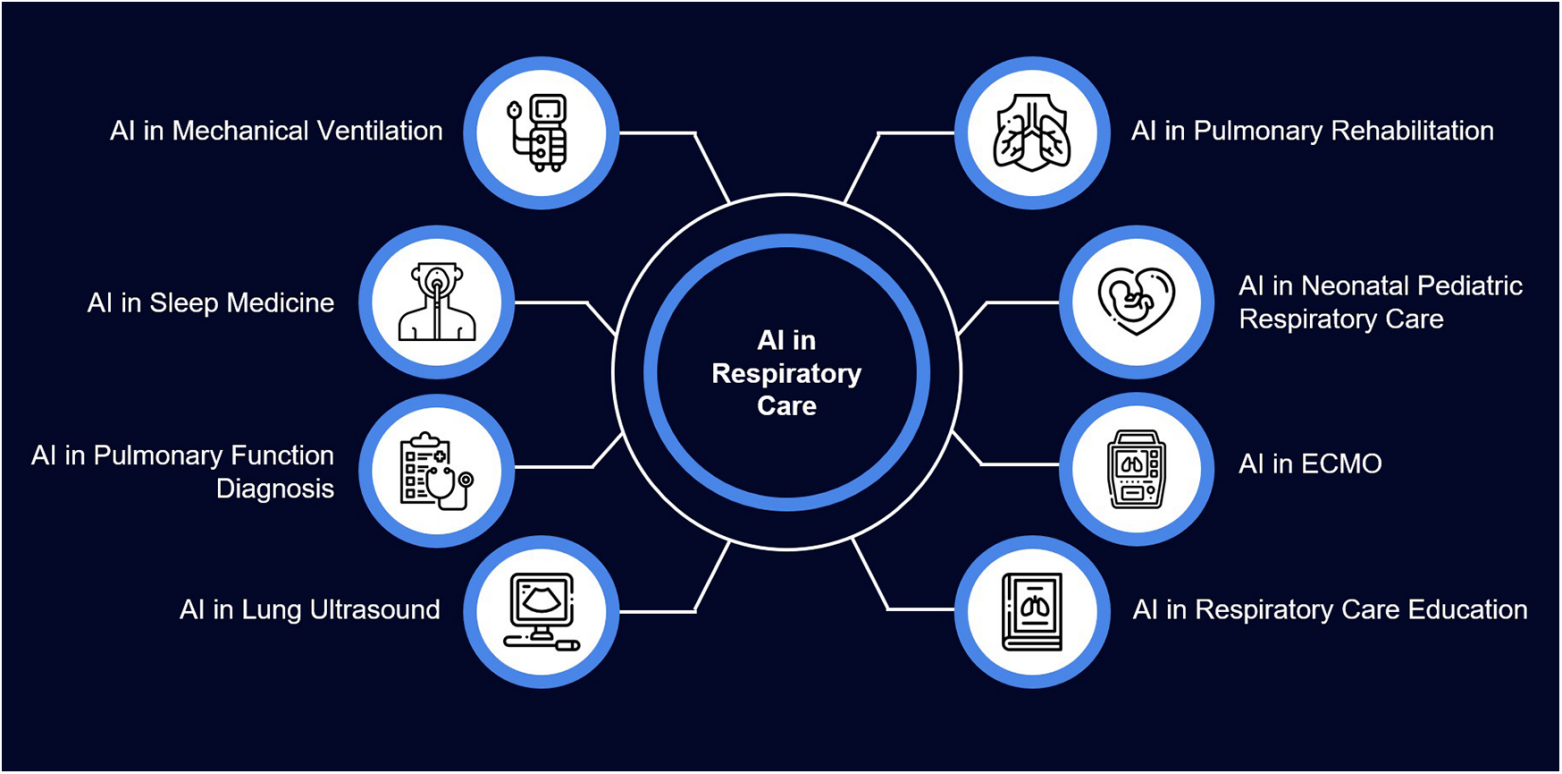
Applications of artificial intelligence in respiratory care.

Apart from these general areas, AI is found to have vast scope in the diagnostic and therapeutic areas of respiratory care (Figure 3). As technology evolves, clinicians must have a broad understanding of its applications.

### AI in mechanical ventilation

Mechanical ventilation is a vital tool used in life-threatening scenarios, making it essential for clinicians to understand its clinical and technical details for safer use. Since the pulmonary mechanics of patients on mechanical ventilation can vary widely, AI can be a useful tool to provide personalised care for each patient and alert clinicians to plan liberation from ventilation.

For example, Peine A et al. (34) developed and evaluated a RL algorithm, VentAI, to suggest a personalised mechanical ventilation regime, focusing on finding the optimal settings for positive end-expiratory pressure, fraction of inspired oxygen, and ideal body weight-adjusted tidal volume. It was noted that the performance of VentAI was significantly superior compared to physicians' standard clinical care, with an observed outcome of in-hospital or 90-day mortality. Rehm GB et al. (35) developed an ML-based classifier to detect abnormal waveform events, specifically injurious subtypes of patient-ventilator asynchronies (double trigger asynchrony and breath stacking asynchrony). The tool accurately detected double trigger asynchrony with a sensitivity of 96% and specificity of 97.5%, and breath stacking asynchrony with a sensitivity of 94.4% and specificity of 98.7%, and other non-patient-ventilator asynchrony events with a sensitivity of 96.7% and specificity of 98%. Adams JY et al. (36) developed an open-source data acquisition system for capturing ventilator waveform data and a modular, multi-algorithm analytic tool (ventMAP) for automated identification of off-target ventilation (OTV) in critically ill patients. The research demonstrated that ventMAP effectively identified harmful effects of OTV, such as excessive tidal volumes and common patient-ventilator asynchronies. Correcting artefacts notably enhanced the specificity of event detection without compromising sensitivity. Sottile PD et al. (37) assessed several ML techniques, including random forest, naïve Bayes, and AdaBoost, on data collected from 62 mechanically ventilated patients who had or were at risk of acute respiratory distress syndrome (ARDS). They chose 116 features based on clinical insight and signal description and were able to determine the presence of synchronous breathing and three types of patient-ventilator asynchrony, including double triggering, flow limited, and ineffective triggering, with an AUC >0.890. Additionally, Loo NL et al. (38) trained a CNN with 5,500 abnormal and normal breathing cycles each, aiming to develop an algorithm to distinguish normal from abnormal breathing cycles, and reported a sensitivity of 96.9% and a specificity of 63.7% for their model.

Another study investigated the scope of AI in predicting prolonged mechanical ventilation and the need for tracheostomy based on severity-of-illness scores calculated from readily available patient variables. The authors concluded that ML predictive models were effective for predicting prolonged mechanical ventilation and tracheostomy placement, with AUCs of 0.82 and 0.83, respectively (39).

Giri J et al. (40) introduced a proposal for a low-cost mechanical ventilator that uses AI technology. The authors suggested a novel design to solve the shortage of ventilators, particularly in resource-limited settings. They used MatLab/Simulink simulation software to develop system-design platforms, employing Mathematical Models to perform simulations under various conditions and parameters. The authors ran computational fluid dynamics simulations to examine the dynamics of airflow, pressure distribution, and gas exchange within the device. These simulations aided in ventilator design and performance optimisation, ensuring the ventilator's efficiency in providing suitable breathing assistance. The authors concluded that the simulation approach using ML was comparable to conventional ventilators, capable of adapting to the patient's breathing patterns, optimising ventilation parameters, and enhancing patient outcomes. Such technological innovations with AI support can be crucial in underserved areas or during emergencies when ventilator demand spikes.

AI has recently demonstrated considerable potential in enhancing the weaning process by predicting the ideal time for weaning and identifying patients who would benefit from weaning. Predicting timely and uneventful liberation from mechanical ventilation has always been of prime interest to respiratory therapists and other intensive care unit (ICU) clinicians. A study that assessed two-stage (time-based) AI prediction models (the try-weaning stage and weaning stage) concluded its effectiveness and precision in predicting the optimal timing to wean intubated patients from the ventilator, with AUCs ranging from 0.843 to 0.953 for stage-1, and 0.889 to 0.944 for stage-2. This approach could reduce patient discomfort, improve medical quality, and lower costs, proving beneficial during pandemics like coronavirus disease 2019 (COVID-19) (41). Smart weaning, an AI-based weaning technique, uses algorithms to modify the ventilator's support according to the patient's respiratory needs. Smart weaning has been shown to enhance patient outcomes, shorten time on mechanical ventilation, and decrease the risk of complications, such as ventilator-associated pneumonia (VAP). Several studies have compared the effectiveness of smart weaning to traditional methods. One study showed that smart weaning was associated with a substantially shorter ventilator duration and lower VAP incidence in a randomised controlled trial of 127 critically ill patients (42). Another study on 240 patients with respiratory failure found that smart weaning had a higher success rate and required less time to perform than traditional methods (43). In the future, it is anticipated that.

AI will be crucial in assisting clinicians in managing ventilated patients. Dual, hybrid, and intelligent ventilator modes are already in place to enhance patient outcomes and lower complications by providing more individualised ventilation using advanced algorithms and patient monitoring. To offer personalized ventilation, dual-mode ventilation integrates inbuilt AI algorithms to combine distinct ventilation techniques, such as pressure support ventilation and volume-controlled ventilation into a single, adaptive system (44). Hybrid mode ventilation enables patients to breathe independently while still receiving ventilator support when necessary, by combining spontaneous breathing and required ventilation (45). Intelligent mode ventilation may reduce the risk of over- or underventilation by adjusting ventilator settings in real-time based on patient requirements and respiratory mechanics using advanced algorithms and monitoring (46). Some literature has reported the relevance of predictive AI models in various aspects of mechanical ventilation. For instance, one group created two models using gradient boosting regression with ICU data, outperforming existing standards with lower errors. Their findings suggested that high average heart rate, diagnosis of respiratory infection, and admissions from locations other than the operating theatre were associated with longer ventilation durations. For non-invasive ventilation, higher respiratory rates and lower Glasgow Coma Scale values were associated with longer durations (47). To err is human, and such errors should not occur with patients on life support devices like mechanical ventilator. Therefore, it is advisable to facilitate and accept technically approved algorithm-based decisions supported by AI programmes, rather than relying solely on human judgments, which are prone to subjective errors.

### AI in extracorporeal membrane oxygenation

Extracorporeal membrane oxygenation (ECMO) is revolutionising critical care as a life-saving therapy for patients with severe, potentially reversible respiratory or cardiac failure (48). AI is now emerging as a transformative tool in ECMO management, aiding in decision-making and prognostication, and optimising clinical outcomes. One key challenge in ECMO management is determining the optimal timing for decannulation. Traditional methods rely heavily on clinical intuition and weaning trials. The Continuous Evaluation of Veno-venous ECMO Outcomes (CEVVO) is a DL-based model designed to predict decannulation success in patients supported on VV-ECMO (49). This model integrates discrete clinical information with continuous data from ECMO devices, employing a long short-term memory-based network to identify temporal patterns. The model categorises patients into risk groups, guiding clinicians on when to initiate weaning trials. The CEVVO model has shown superior performance compared to contemporary models (logistic regression, dense models, decision tree etc) in terms of risk stratification, decannulation outcome and clinical utility, with significantly higher successful decannulation rates in low-risk groups. In venoarterial ECMO (VA-ECMO), predicting patient outcomes is complex due to the high-risk nature of the therapy. The ECMO Predictive Algorithm (ECMO PAL) is the first AI-driven survival score developed using a large international patient cohort (50). Trained on data from the Extracorporeal Life Support Organisation registry, ECMO PAL demonstrated high sensitivity and precision in predicting in-hospital mortality. The model outperformed existing prognostication scores such as ’Survival After Veno-arterial ECMO' (SAVE) and Modified SAVE, highlighting its potential for broader clinical application. Similarly, another study developed an ML model to predict survival to discharge for patients on VA-ECMO, using data from the initial 48 h of support (51). This model, which outperformed the SAVE score, identified key variables, such as lactate, age, total bilirubin, and creatinine as significant predictors of survival, demonstrating the potential of AI to enhance clinical decision-making. Furthermore, AI's role extends beyond ECMO-specific models to broader applications in managing respiratory conditions that may require ECMO. A multimodal AI system was developed to predict the prognosis and required interventions for COVID-19 patients, incorporating clinical findings, laboratory data, and chest x-ray features (52). This system achieved higher predictive accuracy than single-modal models, demonstrating the value of combining different data types for comprehensive prognostication. Such approaches can inform decisions about the necessity of ECMO and other interventions, optimising resource use in critical care settings. Higher-volume ECMO centers have been associated with lower mortality rates, emphasising the importance of experience and expertise in ECMO management (53). AI models can further enhance outcomes in these settings by providing data-driven insights to support clinical decisions. Encouraging the use of AI in high-volume centers could lead to better standardisation and optimisation of ECMO practices. AI and ML are increasingly being explored for their potential to enhance extracorporeal cardiopulmonary resuscitation (ECPR) by optimising patient selection, predicting outcomes, and improving clinical decision-making. AI and ML techniques can analyse large volumes of structured and unstructured data to develop predictive models that identify which patients are most likely to benefit from ECPR. These technologies can also explore treatment heterogeneity, allowing for personalised treatment strategies based on individual patient characteristics (54). Studies have demonstrated that precise patient selection is crucial for achieving better neurological outcomes (55, 56). Additionally, AI can optimise the logistics of ECPR initiation. AI-driven systems can streamline preparation and setup processes, thereby reducing the time to cannulation and initiation of ECMO. Shortened setup times have been associated with improved rates of return of spontaneous circulation and overall survival (57). Moreover, AI can provide real-time monitoring and decision support during ECPR. By continuously analysing physiological parameters, AI systems can assist in adjusting ECMO settings and other supportive measures to optimise patient outcomes. This capability is particularly valuable in managing complex cases where rapid decision-making is critical (58). Finally, AI-driven simulation tools can enhance the training of medical personnel in ECPR procedures. These tools offer realistic scenarios and feedback, thereby improving the proficiency and readiness of the ECPR team (57). Despite the promising applications of AI and ML in ECPR, several challenges need to be addressed to ensure their effective integration into clinical practice.

High-quality, reliable data are essential for developing accurate ML models while using these tools in ECMO patients. Rigorous validation processes are necessary to ensure the reliability of these models. Furthermore, the potential risks of self-fulfilling forecast and feedback loops must be managed carefully.

### AI in neonatal and paediatric respiratory care

AI clinical decision-support tools, though advanced, face implementation challenges due to limited datasets and imbalanced data, particularly in neonatal medicine. Despite these challenges, AI applications in neonatal respiratory care include tools for monitoring vital signs, predicting diseases like respiratory distress syndrome (RDS) and bronchopulmonary dysplasia (BPD), assessing risks such as retinopathy of prematurity, and providing diagnostic and prognostic support for neurological conditions like sleep stage classification.

RDS, common in premature infants due to pulmonary surfactant deficiency, affects over 80% of infants born before 29 weeks gestation (59). Infants with severe RDS are at higher risk of developing BPD (60). Traditional biomarkers like the lecithin/sphingomyelin ratio have limited bedside utility due to technical complexity. Ahmed W et al. (61) explored an ML algorithm to analyse other biomarkers in biological samples, offering a potential bedside tool for clinicians to guide interventions in preterm infants with RDS. Research in paediatric respiratory care includes risk stratification for BPD to identify infants who may benefit from preventive approaches like corticosteroids or patent ductus arteriosus management. The BPD Outcome Estimator, endorsed by the US National Institute of Child Health and Human Development, is widely used for guiding treatment decisions and counselling families (62). However, this tool's scope was limited to neonates of White, Black, or Hispanic descent. Patel M et al. (63) extended this ML algorithm to predict respiratory outcomes in extremely premature infants of Asian heritage, confirming its feasibility. Another study developed an ML algorithm to predict survival without BPD by retrospectively analysing perinatal factors and respiratory support in preterm infants. The combined model, using all perinatal features and 14 days of respiratory data, provided the best prediction with AUCs of 0.921 and 0.899 in the training and testing datasets, respectively. The model also suggested that extubating the patient to CPAP is superior to non-invasive positive pressure ventilation (NIPPV) in BPD-free survival (64). Apnoea of prematurity (AOP) affects over 50% of preterm babies and is nearly universal in infants with extremely low birth weight, linking it to brain damage and poor neurodevelopmental outcomes (65). A ML-based AOP detection model was developed to identify true apnoea in infants by analysing electrocardiographic data. With a false positive rate threshold of 0.3 in the mean receiver operating characteristic curve, the model achieved a high detection accuracy for all cardiorespiratory events (78.2%), and even higher for events followed by bradycardia (93.4%) and desaturation (95.2%). The study concluded that AI-based detection improved apnoea recognition with fewer false alarms than traditional methods (66). Correa M et al. studied 60 lung ultrasound (LUS) frames from 21 children under 5 in Peru to develop a DL algorithm to classify pneumonia. Using neural networks to analyse 1,450 pneumonia vectors and 1,605 normal lung vectors, the authors concluded that the DL model had 100% specificity and 91% sensitivity in diagnosing pneumonia in paediatric inpatients (67). Additionally, AI is used to analyse respiratory sounds in the paediatric population. Techniques like ANN and k-nearest neighbour are successful in recognising and categorising aberrant respiratory sounds (68). AI-based methods for wheeze identification in children and cough sound analysis also enhance diagnostic accuracy (69, 70).

While DL and newer AI algorithms hold significant potential in paediatric respiratory care, further research is needed to fully understand their capabilities. Medical professionals must be trained in AI tool usage, and regulatory procedures must ensure data security and confidentiality in AI applications.

### AI in pulmonary rehabilitation

AI-driven systems in pulmonary rehabilitation utilise wearable devices and sensors to monitor vital parameters like respiratory rate, oxygen saturation, and physical activity levels. These data are crucial for managing conditions such as COPD and interstitial lung disease. By applying ML algorithms, these systems can analyse real-time data to detect anomalies and predict exacerbations, enabling timely interventions and reducing hospital readmissions (71, 72). One study used ML to predict the effectiveness of pulmonary rehabilitation in convalescent COVID-19 patients, based on improved performance in the 6-minute walking test (6MWT). They classified 6MWT performance into three categories (low, medium, and high) and employed various algorithms, including random forest, adaptive boosting, and gradient boosting. Among 189 patients, random forest achieved the highest accuracy (83.7%), sensitivity (84.0%), and AUC (94.5%), while adaptive boosting had the highest specificity (92.7%). The study concluded that ML models are effective in predicting rehabilitation outcomes for convalescent COVID-19 patients (73). AI and ML are pivotal in personalising pulmonary rehabilitation by analysing patient-specific data, such as genetic information, environmental exposures, and health records. These algorithms can identify unique disease phenotypes and predict individual responses to rehabilitation. Personalised rehabilitation protocols can then be optimised to enhance effectiveness and minimise adverse effects. For example, ML models can tailor exercise regimens to match patients' physical capabilities and progression, ensuring safe and effective rehabilitation (74, 75). AI-powered virtual assistants and chatbots are revolutionising patient engagement in pulmonary rehabilitation. These tools utilise NLP technology to provide personalised education, medication and exercise reminders, and real-time symptom management. They also collect patient feedback to refine care plans and improve compliance (76, 77). Predictive analytics driven by AI and ML can forecast disease progression and identify high-risk patients. For instance, RNNs can analyse home spirometry data to predict lung function decline, enabling early intervention. This capability is essential for managing progressive diseases and improving patient outcomes (78).

In conclusion, AI, ML, and DL play a multifaceted role in pulmonary rehabilitation, including diagnostics, personalised treatment, patient engagement, and predictive analytics. These technologies enhance the accuracy and efficiency of pulmonary rehabilitation, empowering patients to actively manage their health and improving clinical outcomes and quality of life for those with chronic respiratory diseases (79).

### AI in lung ultrasound

AI is increasingly used to interpret and analyse CT and x-ray images, employing supervised learning to identify and track respiratory illnesses (80). Over the past two decades, LUS has become a crucial bedside imaging tool in acute care settings (81). AI-enhanced ultrasound machines can now perform complete lung scans in minutes (82). However, less experienced staff may find it challenging to gather footage for analysis, which can limit the utility of automated or AI-driven techniques. Nevertheless, with proper use, even novices can diagnose conditions such as pneumonia, pneumothorax, or effusions using this technology. Recent advances in ML have enabled automated photo recognition to match the accuracy of trained physicians (83). COVID-19 pneumonia often presents with B-line artifacts and pleural line irregularities on LUS, reflecting interstitial thickening and inflammation, which worsens as the disease progresses (84). LUS findings in the anterior lung fields, such as B-lines, consolidation, and pleural thickening, have been associated with the need for intubation in COVID-19 patients (85). Arntfield R et al. (86) developed a DL algorithm trained on a diverse dataset of LUS videos from patients with COVID-19 pneumonia, non-COVID ARDS, and hydrostatic pulmonary oedema, achieving high accuracy in distinguishing these pathologies. Born J et al. (87) introduced a CNN model (POCOVID-Net) to differentiate COVID-19 patients from those with bacterial pneumonia or healthy individuals, achieving an accuracy of 89% and a video accuracy of 92%. Kuroda Y et al. (88) evaluated an AI-based pneumonia detection method using a pocket-sized point-of-care ultrasound (AI-POCUS) device for COVID-19 pneumonia, achieving high sensitivity (92.3%) and specificity (100%). Another group developed a DL algorithm to quantify B-lines in LUS assessments, comparing it with expert human interpretations for both binary and severity classifications. The DL model achieved a sensitivity of 93% and a specificity of 96% for detecting B-lines compared to human experts (89). In a novel DL method called automated M-mode classification, the absence of lung sliding motion was detected with a balanced accuracy of 89%, sensitivity of 82%, and specificity of 92% (90). Another study developed and validated a binary classifier to distinguish between the presence and absence of lung sliding on LUS. Using a labelled dataset of 2,535 clips from 614 patients, the DL binary classifier achieved a sensitivity of 93.5%, specificity of 87.3%, and an AUC of 0.973 (91). Researchers also evaluated a DL algorithm using a spatial transformer network to detect and quantify pleural effusion based on images, finding improved accuracy compared to clinical standards (92).

AI integration in LUS holds great potential; however, robust applications require substantial high-quality data for training. The limited availability of LUS imaging data compared to other widely available imaging modalities poses a challenge to developing these applications.

### AI in sleep medicine

AI is increasingly used to evaluate sleep quality, analysing heart rate, breathing rate, and body movements to provide clinicians with a comprehensive understanding of a patient's sleep, thereby aiding informed treatment decisions (93). AI has the potential to revolutionise sleep medicine by enabling early detection, personalised treatment, and better management of sleep disorders. In conditions like obstructive sleep apnoea (OSA), AI is expanding beyond the traditional apnoea-hypopnoea index for diagnosis (94). While OSA is usually diagnosed using polysomnography—which requires an overnight stay in a sleep lab or home testing—researchers are developing more accessible and affordable methods using AI. For example, smartwatches can monitor blood oxygen levels, providing a tool for early OSA detection (95, 96). AI-based predictive models have also shown promise in identifying individuals at risk of developing OSA using predictors like age, sex, and body mass index (97, 98). Furthermore, AI aids in diagnosing and subtyping narcolepsy by analysing clinical and polysomnographic data (99, 100). Li C et al. (101) proposed a DL model (EEGSNet) using multi-layer CNNs to extract features from EEG and bi-directional long short-term memory networks to classify sleep stages, outperforming other technologies, particularly for the challenging N1 stage. DL algorithms improve the precision and reliability of sleep studies, leading to more accurate diagnosis and treatment of sleep disorders. AI can also track sleep-related movement disorders such as REM sleep behaviour disorder, restless legs syndrome, and periodic limb movement disorder. Diagnosing narcolepsy is difficult due to the variability of multiple sleep latency tests, but an AI algorithm has shown promise in diagnosing type 1 narcolepsy in a single night with reasonable accuracy (102). Furthermore, Brennan HL and Kirby SD (103). in their review concluded with the utility of AI to individualise treatment regimens for sleep disorders. They underline the potential of AI to improve the treatment of OSA through predicting outcomes, and evaluating the treatment based on the disease process and physiology.

AI algorithms can identify patterns in patient data, such as sleep study results, medical history, and lifestyle factors, that may not be evident to physicians. However, clinicians should be aware of potential biases in these applications and the need for human supervision when designing sleep-related diagnostic and therapeutic plans.

### AI in pulmonary function diagnostics

AI has the potential to revolutionise pulmonary function evaluation, enhancing diagnostic precision by improving the interpretation of pulmonary function tests (PFTs) and eliminating inconsistencies in subjective test interpretations among pulmonologists (104). AI-based software has been successfully implemented for the computerised interpretation of PFTs, aiding in diagnosing lung diseases (105). Spirometry remains the most reliable method for detecting obstructive airway diseases, yet its underuse and misinterpretation have led to underdiagnosis (106). The rapid advancement of AI in medicine has improved PFT interpretations. A decision tree model that incorporates lung function and clinical variables has shown improved accuracy in detecting common lung diseases, such as COPD, asthma, interstitial lung disease, and neuromuscular disorders, compared to using PFT data alone (107). The study, which included 968 new patients in a pulmonary practice, demonstrated that the American Thoracic Society (ATS) and European Respiratory Society (ERS) algorithm correctly diagnosed 38% of patients, with COPD having the highest positive predictive value (74%). The decision tree algorithm doubled overall accuracy to 68% and improved positive predictive values for COPD (83%), asthma (66%), interstitial lung disease (52%), and neuromuscular disorders (100%). Another study using a neuro-fuzzy system with spirometric parameters and clinical symptoms achieved over 99% accuracy in classifying asthma and COPD (108). The neuro-fuzzy system integrates neurology-based learning with fuzzy logic's interpretability (e.g., If-Then). Topalovic M et al. (104) compared pulmonologists with AI software in identifying PFT patterns, finding that pulmonologists matched guidelines in about 75% of cases, with a correct diagnosis in 44.6 ± 8.7% of cases. In contrast, the AI software matched PFT pattern interpretations in 100% of cases, with an accurate diagnosis in 82%. Andrade D et al. (109) studied a diagnostic model using ML and the forced oscillation technique to detect respiratory changes in systemic sclerosis patients, concluding that ML algorithms combined with oscillation principles are effective in diagnosing respiratory changes. Another study by Amaral JL et al. (110) classified the severity of airway involvement in COPD using ML algorithms and forced oscillation measures, yielding promising results for precise diagnosis and monitoring. These studies underscore the potential of AI in improving the accuracy and efficiency of PFT interpretation and diagnosis.

### AI in respiratory care education

AI can help reduce the workload for students and teachers while offering more engaging learning opportunities (111). It is expected to tailor learning experiences to individual students and incorporate active learning into education (112). AI can facilitate student learning through direct teaching (acting like a teacher), support teaching (collaborating with students), and empowering the learner (enabling group problem-solving with teacher feedback) (113–115). AI algorithms can analyse individual learning styles, progress, and strengths or weaknesses, creating personalised learning paths to optimise the educational experience. Liventsev V et al. (116) highlighted the importance of AI in healthcare education, particularly in patient simulators. Tools like “Auto-ALS” and GraphSim align with respiratory care curriculum objectives, using reinforcement learning to enhance training (116). AI virtual environments, such as SimX and Body Interact, are crucial in healthcare education, offering immersive, interactive scenarios for students to practise and refine their skills. These platforms provide dynamic, realistic patient interactions, essential for developing critical thinking and decision-making skills, which are vital in respiratory care. Through simulations, students encounter various clinical scenarios without risking real patients, enhancing clinical competence and confidence in handling real-life challenges (117). Adaptive e-learning systems like Firecracker, Smart Sparrow, and Coursera have significantly contributed to medical education by tailoring learning experiences to each student's needs. These platforms adjust content and pace based on individual performance, presenting more challenging cases to those excelling and providing extra resources to those struggling. This personalised approach is crucial in mastering the vast subject matter in medical studies. By continuously analysing performance, these systems optimise the learning journey in real-time, preventing students from feeling overwhelmed or under-challenged (118). In health professions education, AI-driven assessments have transformed student performance evaluations. Platforms such as ExamSoft and Gradescope offer precise, efficient evaluation methods, providing thorough and unbiased assessments. AI swiftly analyses outcomes objectively, delivering timely, precise feedback essential for students to identify strengths and address knowledge gaps, enhancing their learning journey (119). These technologies adapt simulation settings based on individual skill levels (120), ensuring a targeted, efficient educational journey and better translation of theoretical concepts into clinical proficiency (119). Health professions education must adapt to evolving technologies and their ethical implications. As AI brings significant changes, respiratory care professionals in academic and clinical contexts should receive proper training to employ these tools effectively (119, 121).

## Discussion

The application of AI in respiratory care is promising, yet there remain areas that require further research and refinement to fully understand its potential. Though the short-term success of the role of AI in respiratory care tools, such as improved diagnostic accuracy and personalized ventilator management, has been well-documented, there remains a lack of evidence on its utility in the long-term outcomes of respiratory patients. Hence, it is recommended to have predictive AI models capable of analyzing patients' data to assist clinicians with optimal decision-making and to identify the patients at a higher risk of complications, morbidity, or mortality (122).

The evolution of wearable devices such as watches, patches, or devices, digital stethoscopes, smart inhalers, portable smart spirometers, and telemedicine has significantly contributed to the advancement of respiratory care, enhancing both diagnostics and therapeutics. Wearables with AI-integrated sensors and processors facilitate wireless data transmission to clinicians, allowing remote monitoring and assessment of the physical, physiological, and biochemical parameters of patients more accessible (123). While portable smart spirometers facilitate easy, accurate pulmonary function assessment in remote settings, ensuring that patients with COPD and asthma regularly monitored, digital stethoscopes and smart inhalers have improved diagnostic precision and medication adherence, respectively, by integrating AI models with monitoring and feedback systems into conventional tools. These technologies, combined with telemedicine, enable remote consultations, data sharing, and patient education, thereby facilitating remote access to efficient respiratory care (24).

Literature reflects more on the development, validation, and evaluation of AI tools in healthcare; however, there is an observed research gap between the development of robust algorithms and the implementation of AI systems in healthcare practice (124). Although literature reflects the promising potential of AI in enhancing healthcare services, including respiratory care, there remain several challenges that delay its implementation. One of the key challenges includes data protection, privacy, and security concerns, as the development of algorithms based on synthetic data requires sensitive patient information that demands protection against data leaks or misuse. Additionally, the lack of standardized data, interoperability, and variability among healthcare systems make it difficult for the seamless integration of AI algorithms. Another key area of implementation is the acceptance of this technology by healthcare leaders, healthcare professionals, and patients (125). Resistance to changes in the existing system by healthcare leaders and fear of job loss by healthcare professionals are some of the factors that contribute to the slow and variable uptake of this technology in the healthcare system.

To facilitate acceptance from end-users like healthcare professionals, specialty-level benefits of AI should be explained, such as a reduction in fatigue-related human errors, reduced cost, assistance in labour-intensive tasks, less need for invasive procedures, and lowered mortality rates. Moreover, healthcare professionals should be upgraded in their technical and technological skills related to AI for swift adoption. However, besides acquiring AI-related essential skills, it is important that healthcare professionals understand and address the associated queries or ethical and legal issues. A key question, “How can we ensure that AI technology is safe for patients?” is significant because end-users or healthcare professionals may not always be able to provide an explanation due to their lack of expertise in AI-related technological areas (126). Ethical considerations related to the transparency of decision-making processes and high costs associated with the development and deployment of the tools, insufficient infrastructure, and a shortage of skilled personnel further complicate the adoption of this technology.

Therefore, it is most important that key stakeholders work to address the lack of clear regulatory frameworks and guidelines on integrating AI technology into healthcare. Clarifying these uncertainties will facilitate its acceptance and implementation.

Furthermore, the integration of AI technologies into healthcare practices not only enhances patient care but also aligns with the United Nations Sustainable Development Goals (SDGs), highlighting its wider impact on global health and development. For example, the integration of AI in respiratory care aligns with SDG 3 (Good Health and Well-being) by improving patient outcomes through early disease detection and personalized care that includes various technologies in acute care and rehabilitation. The utility of AI-based simulation tools in respiratory care education supports SDG 4 (Quality Education) by preparing competent healthcare graduates. Additionally, the advancement of AI technologies in respiratory care promotes innovation in healthcare infrastructure, aligning with SDG 9 (Industry, Innovation, and Infrastructure) by promoting sustainable technological progress in clinical practices. Lastly, and most importantly, collaborative efforts of respiratory care professionals, medical leadership, AI specialists, and stakeholders in AI research and application support SDG 17 (Partnerships for the Goals) to exchange knowledge and resources, facilitating better patient care, sustainability objectives and improving global health outcomes (127).

## Summary

AI is significantly impacting respiratory care, advancing areas such as mechanical ventilation pulmonary diagnostics, sleep medicine, neonatal and paediatric respiratory care, LUS, pulmonary rehabilitation, and education. AI technologies have transformed patient management by optimising ventilator settings and reducing ventilator-induced lung injuries. AI algorithms analyse real-time data—such as respiratory rate, tidal volume, and airway pressure—providing personalised and adaptive ventilator settings tailored to individual patient needs. Closed-loop systems further enhance outcomes by automatically adjusting ventilator parameters based on continuous feedback, improving patient outcomes and reducing healthcare provider workload. In pulmonary diagnostics, AI enables precise early detection of diseases like COPD and asthma through spirometry data analysis. AI tools interpret complex respiratory patterns, offering diagnostic insights that surpass traditional methods, facilitating timely and accurate clinical decisions. In sleep medicine, AI improves the diagnosis and treatment of disorders like obstructive sleep apnoea by analysing polysomnography data to identify abnormalities and predict patient responses to treatments, enhancing diagnostic accuracy and personalising treatment plans. Neonatal and paediatric respiratory care benefit from AI in monitoring and managing respiratory distress in preterm infants. AI systems predict complications and guide interventions, reducing chronic lung disease and improving survival rates. In LUS, AI enhances the accuracy and efficiency of diagnosing conditions such as pneumothorax, pleural effusion, and interstitial lung disease. Deep learning algorithms quickly interpret ultrasound images, offering real-time diagnostic support to clinicians and speeding up pulmonary assessments. Pulmonary rehabilitation has been revolutionised by AI through personalised exercise regimens and progress monitoring. AI analyses data from wearable devices to customise rehabilitation programmes, optimising outcomes and increasing patient engagement. AI has also advanced respiratory care education by providing simulation platforms for training healthcare providers. These platforms create realistic clinical scenarios, enhancing skills in a controlled environment and ultimately improving clinical competence and patient care standards. A tabular summary on the application, models, advantages and challenges limitations of AI in the field of respiratory care is highlighted in Table 1.

**Table 1:** AI tools, advantages, and limitations/challenges across various domains related to respiratory care.

Area of Application	AI tools/models	Advantages	Limitations/challenges
Mechanical ventilation	VentAI, ML-based classifiers	Personalised care, improved weaning prediction, reduced VAP risk, rapid decision-making	High-quality data requirement, complexity in diverse patient response
Extracorporeal membrane oxygenation	CEVVO, ECMO PAL, ML models for survival prediction	Enhanced decision-making, real-time monitoring, effective ECPR logistics	Data reliability, risks of feedback loops, high-quality data needed
Neonatal & pediatric respiratory care	ML for RDS, BPD Outcome Estimator, DL models for pneumonia	Improved disease prediction, accurate diagnosis, reduced false alarms	Limited datasets, imbalanced data, data confidentiality concerns
Pulmonary rehabilitation	Wearable devices, ML algorithms	Personalised rehab, reduced readmission rates, predictive analytics	Data security, need for personalised exercise regimen training
Lung ultrasound	DL algorithms for pneumonia	Enhanced diagnostic accuracy, faster lung assessments	Limited imaging data, need for skilled personnel for data collection
Sleep medicine	DL models like EEGSNet	Comprehensive sleep evaluation, early detection of sleep disorders	Potential biases, need for human supervision in diagnosis
Pulmonary function diagnostics	Decision tree models, neuro-fuzzy systems	Improved accuracy in disease identification, reduced interpretation inconsistencies	High-quality data requirement, variability in real-world application
Respiratory care education	AI simulators (SimX, Body Interact), adaptive e-learning systems	Personalised learning, critical thinking enhancement, efficient evaluation	Ethical considerations, need for faculty and student training

AI has the potential for improving patient outcomes, enhancing safety, and raising care quality in respiratory diagnostics and therapeutics. However, successful integration of AI into clinical practice depends on healthcare professionals' acceptance and understanding of the technology. Establishing clear, widely agreed-upon standards communicated in accessible language is crucial. This approach will facilitate AI adoption in respiratory care, ensuring its benefits are realised while maintaining high standards of patient-centred, evidence-based practice, and contributing to improved clinical outcomes.
